# Therapeutic Effect of Eugenol‐Loaded Chitosan Nanoparticles Against *Helicobacter pylori* Infection: A Histologic and Molecular Study

**DOI:** 10.1155/ijbm/6699913

**Published:** 2026-04-01

**Authors:** Mohammad Javad Tahmasebi, Mahmoud Osanloo, Alireaza Tavassoli, Abbas Abdollahi, Elham Zarenezhad, Fatemeh Norouzi, Behnoosh Miladpour

**Affiliations:** ^1^ Student Research Committee, Fasa University of Medical Sciences, Fasa, Iran, fums.ac.ir; ^2^ Department of Medical Nanotechnology, School of Advanced Technologies in Medicine, Fasa University of Medical Sciences, Fasa, Iran, fums.ac.ir; ^3^ Department of Pathology, School of Medicine, Fasa University of Medical Sciences, Fasa, Iran, fums.ac.ir; ^4^ Department of Microbiology, School of Medicine, Fasa University of Medical Sciences, Fasa, Iran, fums.ac.ir; ^5^ Noncommunicable Diseases Research Center, Fasa University of Medical Sciences, Fasa, Iran, fums.ac.ir; ^6^ Department of Clinical Biochemistry, School of Medicine, Fasa University of Medical Sciences, Fasa, Iran, fums.ac.ir

**Keywords:** caspase-3, chitosan nanoparticles, eugenol, *H. pylori*, IL-1β, IL-8, TNF-α

## Abstract

**Background:**

Eugenol is a phenolic compound known for its antimicrobial, antifungal, and antioxidant properties. Its encapsulation in chitosan nanoparticles (EuChiNPs) enhances stability and therapeutic efficacy.

**Objectives:**

This study aimed to investigate the effects of EuChiNPs on *Helicobacter pylori (H. pylori*) infection in rats, focusing on its impact on inflammatory and apoptotic pathways.

**Methods:**

EuChiNPs were synthesized and characterized using DLS, TEM, and FTIR techniques. A total of 42 male Wistar rats were divided into six groups, including controls, *H. pylori*‐infected, and treatment groups receiving EuChiNPs, standard antibiotics, or a combination. The effects of treatments were evaluated using RT‐PCR, ELISA, Western blotting, and histopathology.

**Results:**

The combination of EuChiNPs and standard antibiotics significantly reduced the expression of inflammatory markers interleukin‐1 beta (IL‐1β) and interleukin‐8 (IL‐8). Serum levels of IL‐1β, IL‐8, and TNF‐α were markedly decreased in the combination group compared with antibiotics alone (*p* < 0.0001). Western blot analysis revealed a reduction in caspase‐3 and caspase‐7 expression, indicating attenuation of apoptosis. Histopathological analysis showed improved mucosal integrity and reduced bacterial density in the combination treatment group.

**Conclusion:**

EuChiNPs demonstrated strong anti‐inflammatory and antiapoptotic effects, especially when combined with standard antibiotics. This novel approach promises to improve *H. pylori* eradication while minimizing antibiotic resistance and side effects. Further clinical studies are recommended to validate these findings.

## 1. Background

*Helicobacter pylori* (*H. pylori*), first identified in 1982, is a Gram‐negative, flagellated, and microaerophilic bacterium that colonizes the human gastric mucosa. It is a significant public health concern, infecting over half of the global population and contributing to various gastrointestinal diseases, including chronic gastritis, peptic ulcers, and gastric cancer [1]. The bacterium’s pathogenicity is primarily attributed to its ability to produce urease, disrupt gastric acid secretion, and induce inflammatory responses mediated by cytokines such as interleukin‐1 beta (IL‐1β) and tumor necrosis factor‐alpha (TNF‐α). These inflammatory mediators are critical in gastric mucosal damage and disease progression [2–4]. The treatment of *H. pylori* infections faces considerable challenges due to the increasing prevalence of antibiotic resistance, particularly to clarithromycin and levofloxacin. This resistance has undermined the efficacy of standard eradication therapies, often necessitating the use of aggressive and costly quadruple regimens. Moreover, the extensive use of antibiotics during the COVID‐19 pandemic has further exacerbated this issue, highlighting the urgent need for novel therapeutic strategies [5–8]. The treatment of *H. pylori* infections faces significant challenges primarily due to the rising prevalence of antibiotic resistance. Resistance to commonly used antibiotics such as clarithromycin, metronidazole, and levofloxacin has considerably reduced the effectiveness of standard eradication therapies. This has led to increased treatment failures, requiring more complex and prolonged regimens, which in turn cause higher costs, greater side effects, and lower patient compliance. Additionally, the bacterium’s ability to colonize the gastric mucosa and evade immune responses, along with its intracellular localization, makes it difficult for antibiotics to fully eradicate the infection. These challenges highlight the urgent need for alternative therapeutic strategies that can enhance efficacy and overcome resistance [9]. Eugenol, a naturally occurring phenolic compound derived from clove oil, has garnered attention for its wide range of biological activities, including antimicrobial, antioxidant, anti‐inflammatory, and anticancer properties [8]. Despite its therapeutic potential, the clinical application of eugenol is limited by its volatility, low bioavailability, and potential toxicity at high concentrations [10, 11]. To overcome these limitations, encapsulating eugenol in chitosan nanoparticles (EuChiNPs) offers a promising approach. Chitosan, a biocompatible and biodegradable polymer, enhances eugenol’s stability and controlled release, ensuring its targeted delivery and reducing systemic toxicity [11–13]. Chitosan, a natural cationic biopolymer derived from the deacetylation of chitin, exhibits excellent biocompatibility, biodegradability, and antimicrobial properties, making it a promising material in biomedical applications. Its nanoparticle formation ability enhances drug stability, bioavailability, and targeted delivery, especially in combination therapies. Chitosan nanoparticles (ChiNPs) interact with bacterial membranes through their positive charge, disrupting the cell wall and exhibiting antimicrobial effects, as shown in previous studies involving *H. pylori* treatment [13]. Moreover, ChiNPs improve therapeutic efficacy by protecting encapsulated agents like eugenol from degradation and providing controlled release. These properties position chitosan as an effective carrier for natural compounds, reducing reliance on conventional antibiotics and mitigating resistance issues [13–15]. Given the increasing challenges of antibiotic resistance in the treatment of *Helicobacter pylori* infections, the use of eugenol encapsulated in chitosan nanoparticles (EuChiNPs) can be considered a novel and promising strategy. These nanoparticles improve the stability, bioavailability, and targeted delivery of eugenol, enhancing its antibacterial efficacy and producing a significant synergistic effect when combined with conventional antibiotics. This approach enables reduction in antibiotic dosages, potentially mitigating the development of drug resistance. Additionally, the mucoadhesive properties of ChiNPs facilitate better penetration and prolonged retention in the gastric mucosa, overcoming the limited accessibility of antibiotics to bacterial reservoirs. Furthermore, the anti‐inflammatory properties of eugenol contribute to gastric tissue protection and inflammation reduction, which play important roles in improving therapeutic outcomes. Therefore, EuChiNPs represent an innovative adjunctive approach with the potential to bridge current treatment gaps in *H. pylori* infection, helping to control antibiotic resistance and enhance gastric mucosal health. Despite the well‐documented antimicrobial and anti‐inflammatory properties of eugenol, its direct clinical application remains limited due to volatility, poor bioavailability, and potential toxicity at higher concentrations. On the other hand, current antibiotic regimens for *H. pylori* have shown declining efficacy as resistance to commonly used drugs such as clarithromycin, metronidazole, and levofloxacin continues to rise. While ChiNPs have been explored as drug delivery systems, very few studies have specifically investigated the encapsulation of eugenol in chitosan nanoparticles (EuChiNPs) for in vivo treatment of *H. pylori* infection. Moreover, the molecular mechanisms underlying their therapeutic effects—particularly their influence on inflammatory cytokines (IL‐1β, interleukin‐8 (IL‐8), and TNF‐α) and apoptotic pathways (caspase‐3 and caspase‐7)—remain poorly understood. Importantly, whether EuChiNPs can act synergistically with conventional antibiotics to reduce drug dosage, overcome resistance, and minimize side effects has not been systematically evaluated. These critical gaps highlight the need for novel adjunctive strategies, such as EuChiNPs, to improve the treatment of *H. pylori* infection [13, 14, 16]. This study introduces a novel therapeutic strategy by combining EuChiNPs with standard antibiotic therapy to combat *H. pylori* infection. To the best of our knowledge, this is among the first studies to evaluate the dual role of EuChiNPs in enhancing antibacterial efficacy and modulating inflammatory and apoptotic responses in vivo. By leveraging the synergistic effects of eugenol and chitosan, our approach addresses two major challenges in *H. pylori* treatment: rising antibiotic resistance and the limitations of conventional regimens. The findings of this study may pave the way for the development of more effective, affordable, and sustainable treatments for *H. pylori*‐associated diseases.

## 2. Materials and Methods

### 2.1. Chemicals

The following reagents were used: eugenol (CAS No. 97‐53‐0), chitosan (CAS No. 9012‐76‐4), tripolyphosphate (TPP, CAS No. 7758‐29‐4), chloroform, glycine (CAS No. 56‐40‐6), cDNA synthesis kit, primary antibodies for caspase‐3 and caspase‐7, secondary antibodies (Abcam, UK), ECL kit (Pars Toos, Iran), acrylamide (CAS No. 79‐06‐1), bis‐acrylamide (CAS No. 110‐26‐9), Coomassie Blue (CAS No. 6104‐58‐1), nitrocellulose paper, TRIzol reagent (Product No. T9424, Sigma‐Aldrich, USA), SYBR Green, RIPA buffer, Tris base, nonfat dry milk, methanol, and ELISA kits for rat IL‐8 (Catalog No. KPG‐RIL8), IL‐1β (Catalog No. KPG‐RIL1), and TNFα (Catalog No. KPG‐RTNF).

### 2.2. Instruments and Equipment

The instruments and equipment employed in this study are summarized in Table 1.

**Table 1:** A summary of the instruments and equipment used in this study.

Instrument/apparatus	Model	Manufacturer	Purpose
Dynamic light scattering (DLS)	Zetasizer Nano ZS	Malvern Instruments, UK	Nanoparticle size analysis
Fourier‐transform infrared spectroscopy (ATR‐FTIR)	Nicolet iS50	Thermo Fisher, USA	Nanoparticle characterization
Transmission electron microscopy (TEM)	JEM‐1400	JEOL, Japan	Morphology analysis
Optical microscope	BX53	Olympus, Japan	Histological assessment
Nanodrop spectrophotometer	NanoDrop 2000	Thermo Fisher, USA	Protein and RNA quantification
Real‐time PCR system	StepOnePlus	Applied Biosystems, USA	Gene expression analysis
Autoclave	—	—	Sterilization of media
Microwave oven	—	—	Silver staining

### 2.3. Characterization of Nanoparticles

A 5% w/v chitosan solution was prepared in 1% acetic acid. Eugenol (10 mg) was dissolved in 1 mL Tween‐20 and stirred at 2000 rpm for 15 min. Then, 0.5% chitosan solution (5 mL) and TPP (2 mL, 0.1% w/v) were added dropwise under continuous stirring at room temperature for 30 min. Nanoparticles were characterized using the following analyses.

#### 2.3.1. Particle Size Analysis

The particle size distribution of the formulations was determined using dynamic light scattering (DLS). The SPAN value (d90‐d10/d50) was calculated to assess the size distribution uniformity, with SPAN values below 1 indicating narrow distributions.

#### 2.3.2. The Attenuated Total Reflection‐Fourier Transform Infrared (ATR‐FTIR) Analysis

FTIR spectra were obtained to confirm the successful loading of eugenol into the nanoparticles. Peaks corresponding to characteristic functional groups of eugenol and chitosan were analyzed.

 DLS: Zetasizer Nano ZS (Malvern Instruments, UK).
 FTIR: ATR‐FTIR, Nicolet iS50 (Thermo Fisher, USA).
 TEM: JEOL JEM‐1400 (Japan).

All formulations were prepared and measured in triplicate (*n* = 3). Formulations were abbreviated as ChiNPs (chitosan nanoparticles), EuChiNPs (eugenol‐loaded chitosan nanoparticles), and fChiNPs (free chitosan nanoparticles).

### 2.4. Animal Model and Ethical Statements

All animal experiments were conducted in accordance with national and institutional guidelines for the care and use of laboratory animals. The study protocol was approved by the Institutional Animal Ethic Committee IR.FUMS.AEC.1401.001. All procedures were aligned with accepted ethical principles, including those outlined in Declaration of Helsinki, and every effort was made to minimize animal discomfort and to use the minimum number of animals required.

The sample size was determined based on statistical calculation; 42 healthy male Wistar rats (8–12 weeks old, average weight: 298 g) were housed in a temperature‐ and light‐controlled environment (12 h light/dark cycle) with free access to food and water. According to the findings of a previous study (PMID: 29386977), the maximum statistical power in ANOVA analyses is typically achieved when the degrees of freedom (df) fall within the range of 10–20. Given that the df are calculated as dH.pf = *k*(*n* − 1)df = *k*(n − 1)df = *k*(*n* − 1), where *k* is the number of groups and *n* is the number of subjects per group, we aimed to align our study design accordingly. With six experimental groups (*k* = 6), the optimal statistical power was reached when each group included 4‐5 mice. To ensure robustness, one additional mouse per group was included to confirm the successful establishment of the *H. pylori* infection model, and another was added to account for potential model failures or preventive intervention failures. Three days prior to bacterial inoculation, the rats in the infected groups were administered streptomycin (5 mg/mL) in tap water to suppress the growth of other bacteria. All rats were bred and obtained from the animal lab of Fasa University of Medical Sciences.

The rats were randomly divided into six groups of seven as follows.

1. Control Group (Negative): no infection, no treatment.
2. Control + Nano Group: no infection, treated with 10 mg/kg (according the previous studies’ [17, 18] body weight of EuChiNPs).
3. Positive Control Group (*H. pylori*): infected with *H. pylori* (5 × 10^9^–5 × 10^10^ CFU/mL^−1^ in normal sailin), no treatment.
4. *H. pylori* + Nano Group: Infected with *H. pylori* (5 × 10^9^–5 × 10^10^ CFU/mL^−1^ in normal sailin), treated with 10 mg/kg body weight EuChiNPs.
5. *H. pylori* + Antibiotics Group: infected with *H. pylori* (5 × 10^9^–5 × 10^10^ CFU/mL^−1^ in normal sailin), treated with a standard antibiotic regimen (amoxicillin 50 mg/kg, clarithromycin 25 mg/kg, and omeprazole 20 mg/kg).
6. *H. pylori* + Antibiotics + Nano Group: infected with *H. pylori* (5 × 10^9^–5 × 10^10^ CFU/mL^−1^ in ml normal sailin), treated with 10 mg/kg body weight EuChiNPs and the standard antibiotic regimen (amoxicillin 50 mg/kg, clarithromycin 25 mg/kg, and omeprazole 20 mg/kg).

The treatment dose was selected based on previously published studies that evaluated eugenol against *H. pylori*. Specifically, we referred to studies that demonstrated effective antimicrobial activity within a comparable concentration range without inducing cytotoxicity. The chosen dose falls within the range reported to be both effective and safe in vitro. Relevant references have been cited in the revised manuscript [19, 20]. The groups were treated for 4 weeks and then the rats were anesthetized using injection of a combination of ketamine/xylazine. After anesthesia, cardiac puncture was performed to collect blood, and the serum was separated and stored at −70°C, for future use. Following confirmation of the animal’s euthanasia, the brain, heart, kidneys, spleen, pancreas, liver, and intestines were harvested and preserved at −70°C for subsequent analyses.

### 2.5. Experimental Procedures

#### 2.5.1. *H. pylori* Culture

Columbia agar medium (21 g in 450 mL distilled water) was prepared, sterilized in an autoclave, and cooled to 50°C. Strained egg yolk (50 mL) and antibiotics (vancomycin 10 mg/L, amphotericin B 2.5 mg/L, and trimethoprim 5 mg/L) were added under sterile conditions. Plates were incubated at 37°C for 24 h. *H. pylori* was cultured from gastric biopsy samples obtained from a duodenal ulcer patient at the Pasteur Institute of Iran. Colony morphology, Gram staining, and biochemical tests (oxidase, catalase, and urease) were performed to confirm bacterial identity. Experiments were performed in triplicate (*n* = 3).

#### 2.5.2. Bacterial Inoculation

Rats were pretreated with streptomycin (5 mg/mL) for 3 days before *H. pylori* inoculation to suppress other bacterial growth and then 5 × 10^9^–5 × 10^10^ CFU/mL^−1^ in ml was used for infection inoculation, equivalent to 2.0 McFarland standard in 0.9% NaCl in 1 mL per rat were gavage for 3 days (2 times per day with an interval of 4 h per day). The infected groups received *H. pylori* suspensions, while the control groups received sterile water.

#### 2.5.3. ELISA for Inflammatory Markers

Stomach tissues were homogenized in RIPA buffer, centrifuged, and supernatants were collected. IL‐1β, IL‐8, and TNF‐α levels were measured using ELISA kits (Pars Toos, Iran) according to the manufacturer’s instructions. Protein concentrations were measured using NanoDrop 2000 (Thermo Fisher, USA). All measurements were performed in triplicate (*n* = 3).

### 2.6. Histological Analysis

#### 2.6.1. Hematoxylin and Eosin (H&E) Staining

Tissue sections were deparaffinized, rehydrated through ethanol series (100%, 95%, and 70%), stained with hematoxylin for 10 min, rinsed, treated with 1% HCl and 1% ammonia water, stained with eosin for 5–10 min, dehydrated, cleared with xylene, and mounted. Microscopy was performed using Olympus BX53 (Japan).

#### 2.6.2. *H. pylori* Silver Staining (HpSS)

Sections were deparaffinized, treated with 1% AgNO_3_, irradiated in a microwave, washed, stained with silver solution (gelatin + formic acid), followed by sodium thiosulfate, counterstained with hematoxylin, and examined under a microscope.

### 2.7. Western Blotting

Gastric tissue lysates (20 μg protein) were separated on 12.5% SDS‐PAGE gels and transferred onto nitrocellulose membranes. Membranes were incubated overnight at 4°C with primary antibodies (caspase‐3 and caspase‐7; Abcam, UK), followed by secondary antibodies. Bands were visualized using ECL kit (Pars Toos, Iran) and quantified using ImageJ software.

### 2.8. Real‐Time Polymerase Chain Reaction (RT‐PCR)

Total RNA was extracted using TRIzol (Sigma‐Aldrich, USA) and reverse‐transcribed to cDNA (cDNA synthesis kit). RT‐PCR was performed with SYBR Green on StepOnePlus (Applied Biosystems, USA). Reactions contained 10 μM primers, 25 nM ROX, and 5X Taq polymerase, in a final volume of 25 μL. Cycling: 95°C for 10 min (initial denaturation), 44 cycles at 95°C for 10 s, 55°C for 60 s, and 72°C for 45 s. Gene expression was normalized to GAPDH using the 2^−ΔΔCt^ method (Table 2).

**Table 2:** Primer sequences for IL‐1β and IL‐8.

Gene	Forward (5′ ⟶ 3′)	Reverse (5′ ⟶ 3′)
IL‐1β	CAC​CTC​TCA​AGC​AGA​GAG​CAC​A	GGG​TTC​CAT​GGT​GAA​GTC​AAC​T
IL‐8	AGA​CAG​CAG​AGC​ACA​CAA​GC	CCT​TGG​CAA​AAC​TGC​ACC​TTC​A
GAPDH	GATTTGGCCGTATCGGAC	GAAGACGCCAGTAGACTC

### 2.9. Statistical Analysis

Data are presented as the mean ± SD. One‐way ANOVA followed by Tukey’s post hoc test (GraphPad PRISM 9) was used; *p* < 0.05 was considered significant.

## 3. Results

### 3.1. Characterization of Size and Zeta Potential and Transmission Electron Microscopy (TEM)

**Figure 1:**
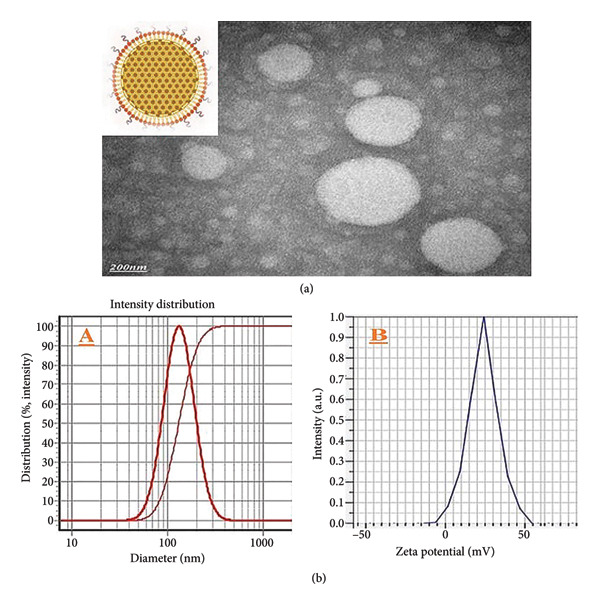
(a) TEM image showing the round shape of eugenol nanoparticles with CH and TPP and (b) DLS nanoparticle size distribution. (A) The mean diameter was 133 ± 80 nm, with a SPAN value of 0.96. (B) The zeta potential of the nanoparticles was measured at +23.8 Mv.

DLS analysis revealed that the average particle size of EuChiNPs was 133 ± 80 nm, with a SPAN value of 0.96, indicating a narrow size distribution. The zeta potential of the nanoparticles was measured at +23.8 mV, confirming their stability. TEM images further confirmed the uniform spherical morphology of the nanoparticles (Figure 1).

### 3.2. Successful Loading of Eugenol in ChiNPs (ATR‐FTIR)

**Figure 2:**
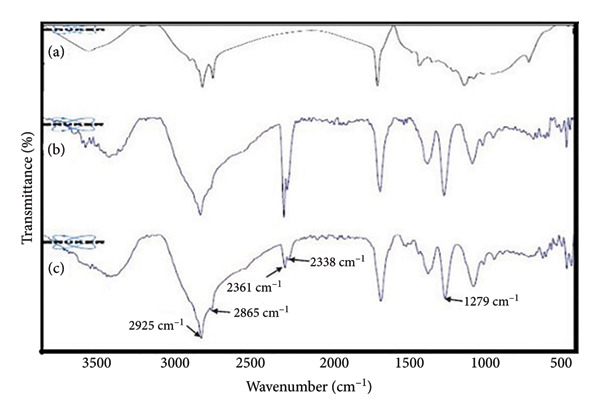
Attenuated total reflectance‐Fourier transform infrared: (a) chitosan, (b) eugenol, and (c) EuChiNP.

FTIR analysis confirmed the successful encapsulation of eugenol in the ChiNPs. Characteristic peaks associated with eugenol (C–H stretching at 2925 and 2865 cm^−1^) and chitosan (C–N stretching at 1279 cm^−1^) were evident, indicating the formation of a stable complex (Figure 2).

### 3.3. Calibration Curve of Eugenol and Encapsulation Efficiency

The calibration curve of eugenol was constructed by measuring the absorbance of standard eugenol solutions at concentrations of 5, 10, 15, 20, 25, and 30 μg/mL in methanol at 282 nm using a UV–Vis spectrophotometer. A strong linear relationship was observed between concentration and absorbance. The regression equation was determined to be

(2) Ŷ=0.02430.0005−,

where *x* represents the eugenol concentration and *y* the corresponding absorbance. The coefficient of determination was calculated using the following formula:

(3) R2=1–Σyi–Ŷ2ΣYi−y®2.

Based on the experimental data, the *R* ^2^ value was found to be 0.998, indicating excellent linearity and reliability of the standard curve.

To calculate the encapsulation efficiency of eugenol in ChiNPs, a known amount of nanoparticles was dissolved in methanol and centrifuged to separate the free eugenol. The absorbance of the supernatant was measured, and the amount of free eugenol was determined using the calibration curve.

Encapsulation efficiency was calculated using the following equation:

(4) Encapsulation efficiency %=total eugenol−free eugenoltotal eugenol×100.

**Figure 3:**
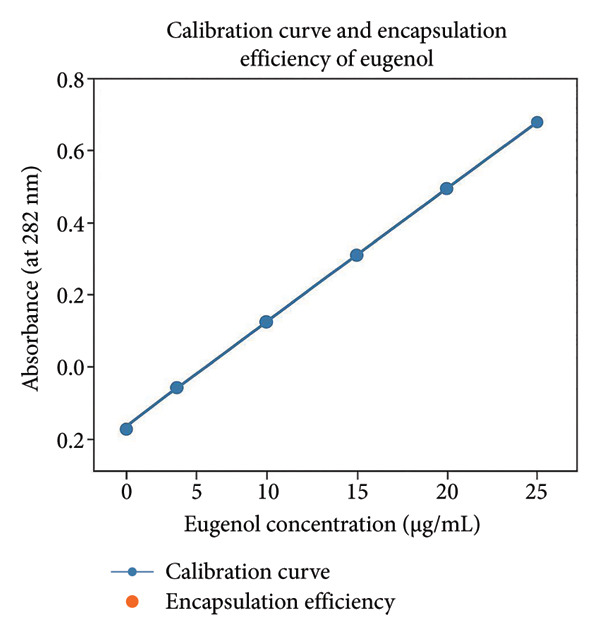
Calibration curve and encapsulation efficiency of eugenol.

In this study, the total amount of eugenol used was 10 mg, and the amount of free eugenol measured was 2 mg. Therefore, the encapsulation efficiency was calculated as follows (Figure 3).

(5) EE%=102−10∗10080=%.

### 3.4. Reduction in Inflammatory Markers

**Figure 4:**
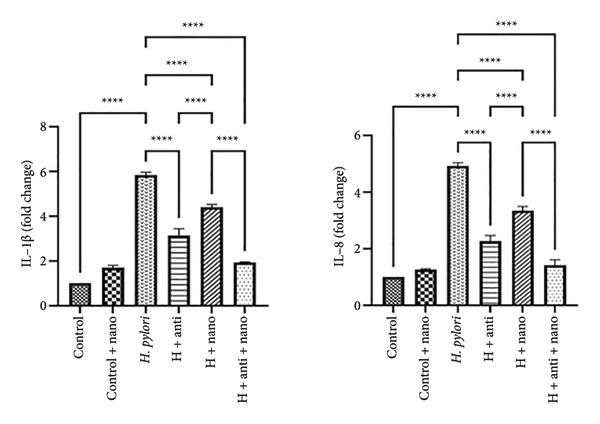
Comparison of the effect of treatment with nanoparticles and standard antibiotic treatment and the mutual effect of these two treatments on the level of IL‐1β and IL‐8 gene expression. IL‐1β in the groups of control (1.00 ± 0.11 times), in the group of *H. pylori* (5.89 ± 0.06 times), in the group of *H. pylori* + nano group (4.40 ± 0.13), in the group of *H. pylori* + antibiotic (2.32 ± 0.33), and in the group of *H. pylori* + anti + nano group (1.93 ± 0.27) and interleukin 8 in the group of control (1.00 ± 0.11 times), in the group of *H. pylori* (4.93 ± 0.12 times), in the group of *H. pylori* + nano group (3.34 ± 0.04), in the group of *H. pylori* + antibiotic (2.12 ± 0.43), and in the group of *H. pylori* + anti + nano group (1.41 ± 0.08) (*p* value: 0.0001 ≥ (^∗∗∗∗^), 0.001 ≥ (^∗∗∗^), (^∗∗^) ≥ 0.01, and (^∗^) ≥ 0.05). One‐way ANOVA.

RT‐PCR analysis demonstrated that the expression levels of IL‐1β and IL‐8 were significantly reduced in the EuChiNPs + antibiotics group compared with the *H. pylori*‐infected control group. Specifically, IL‐1β expression decreased from 5.89 ± 0.06‐fold in the *H. pylori* group to 1.93 ± 0.27‐fold in the EuChiNPs group and 1.41 ± 0.08‐fold in the combination treatment group (*p* < 0.0001). Similar trends were observed for IL‐8 expression (from 4.93 ± 0.12 times in the *H. pylori* group to (3.34 ± 0.04) fold in the EuChiNPs group and 4.93 ± 0.12 times in the combination treatment group) (Figure 4).

**Figure 5:**
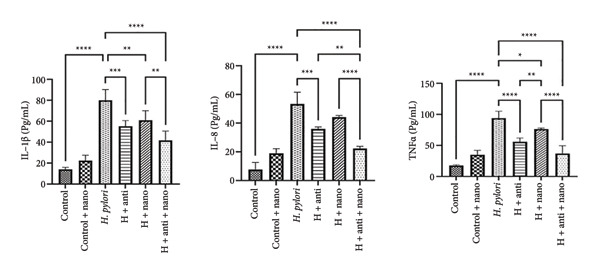
Expression level of IL‐8, IL‐1β, and TNF‐α secretory protein after treatment with EuChiNP and standard antibiotic treatment in the studied groups (*p* value: 0.001 ≥ (^∗∗∗^), 0.0001 ≥ (^∗∗∗∗^), (^∗∗^) ≥ 0.01, and (^∗^) ≥ 0.05). One‐way ANOVA.

As measured by ELISA, serum concentrations of IL‐1β, IL‐8, and TNF‐α also showed a significant reduction in the EuChiNPs and combination treatment groups compared with the antibiotic‐only group, suggesting enhanced anti‐inflammatory effects (Figure 5).

### 3.5. Modulation of Apoptotic Pathways

**Figure 6:**
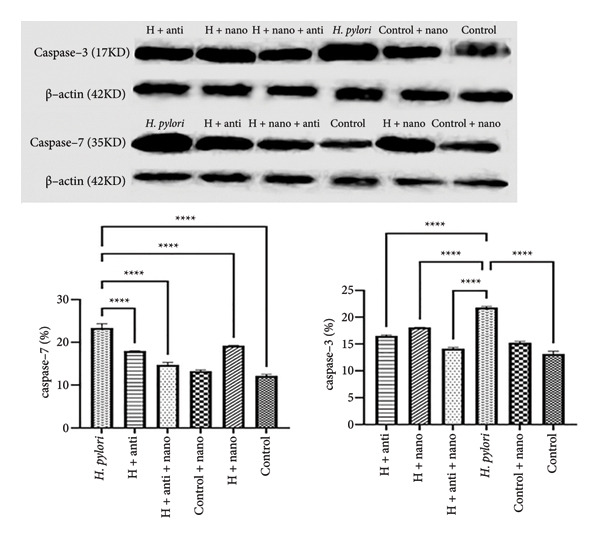
Western blot images and charts to examine caspase‐3 and caspase‐7 proteins in the six studied groups and quantitative Western blot plots (Image J) (*p* value: 0.001 ≥ (^∗∗∗^), 0.0001 ≥ (^∗∗∗∗^), (^∗∗^) ≥ 0.01, and (^∗^) ≥ 0.05). One‐way ANOVA.

Western blot analysis revealed decreased expression of caspase‐3 and caspase‐7 proteins in the EuChiNPs + antibiotics group compared with the *H. pylori*‐infected control group. This reduction in apoptotic markers indicates a protective effect of the nanoparticles against *H. pylori*‐induced gastric mucosal damage (Figure 6).

### 3.6. Histological Findings

**Figure 7:**
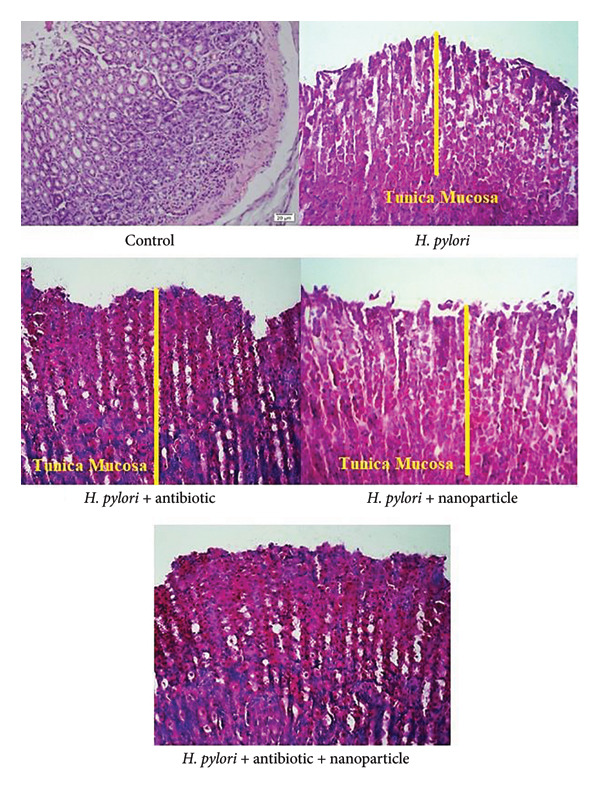
H&E staining. The histological results and thickness of the tunica mucosa in H&E staining (μm) in the groups of control (506 ± 18), in the group of *H. pylori* (305 ± 18), in the group of *H. pylori* + nano group (337 ± 18), in the group of *H. pylori* + antibiotic (335 ± 18), and in the group of *H. pylori* + anti + nano group (477 ± 18), which shows the increase in thickness in the treated group. The number of bacteria in silver staining (mm^2^), in the groups of control (0), in the group of *Helicobacter pylori* (317 ± 10), in the group of *H. pylori* + Nano (224 ± 10), in the group of *H. pylori* + antibiotic (161 ± 10), and in the group of *H. pylori* + nano + antibiotic (34 ± 10).

**Figure 8:**
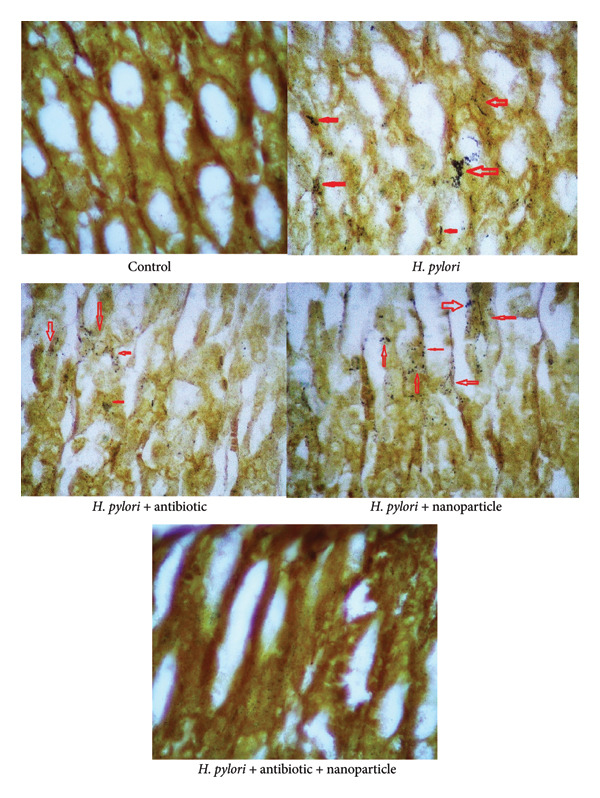
Silver staining. The number of bacteria in silver staining (mm^2^), in the groups of control (0), in the group of *Helicobacter pylori* (317 ± 10), in the group of *H. pylori* + nano (224 ± 10), in the group of *H. pylori* + antibiotic (161 ± 10), and in the group of *H. pylori* + nano + antibiotic (34 ± 10). The red arrows point to the *Helicobacteria pylori*.

**Figure 9:**
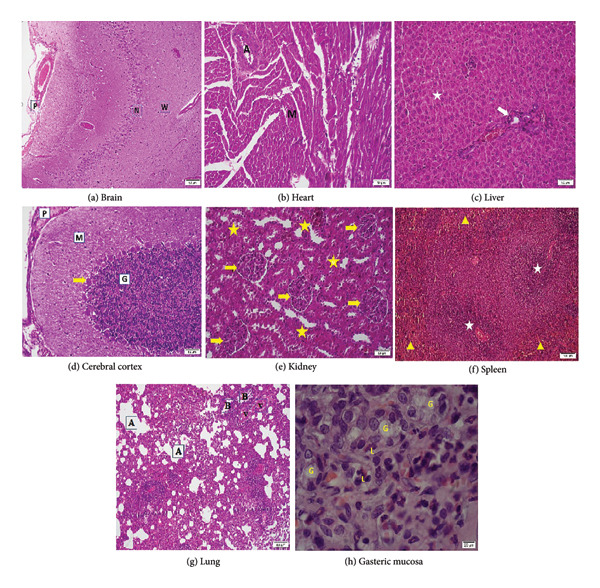
Images of H&E staining of various tissues revealed no pathological abnormalities following treatment with eugenol‐loaded chitosan nanoparticles. (a) The brain, × 200, H&E, Pia mater (P), large cortical neurons (N), and subcortical white matter (W) labeled. (b) Myocardial layer × 200, H&E, many myocardial cells and an arterial vessel (A) labeled. (c) Liver, × 200, H&E, normal hepatocytes in hepatic lobule labeled by white star, and portal triad labeled by white arrow. (d) Cerebellum cortex layers, × 200, H&E, covering pia mater (P), molecular layer (M), granular layer (G), and Purkinje cell bodies (yellow arrow) labeled. (e) Kidney, × 100, H&E, normal glomeruli labeled by arrow and normal areas of tubules and scanty interstitial tissue labeled by star. (f) Spleen, × 100, H&E, white pulp labeled by star and red pulps by yellow arrow head. (g) Lung, postmortem collapsed, × 100, H&E, alveoli (A), bronchioles (B), and vessels (V) labeled. (h) Gastric mucosa, × 1000, H&E, the glands (G), and a few leukocytes in interstitial space (L) labeled.

Histopathological analysis of gastric tissues demonstrated significant improvements in the EuChiNPs and combination treatment groups. H&E staining showed an increase in the thickness of the tunica mucosa (477 ± 18 μm in the combination treatment group vs. 305 ± 18 μm in the *H. pylori* group). Silver staining confirmed a marked reduction in bacterial density, with the number of bacteria decreasing from 317 ± 10 mm^2^ in the *H. pylori* group to 34 ± 10 mm^2^ in the combination treatment group (Figures 7 and 8). After treatment with EuChiNPs, these tissues were analyzed to evaluate the pathological abnormalities in the brain, kidney, spleen, liver, cerebral cortex, heart, heart, lung, and pancreas; these tissues were analyzed followed by H&E staining (Figure 9).

## 4. Discussion

This study evaluated the therapeutic potential of EuChiNPs in treating *H. pylori* infections. Our findings revealed that combining EuChiNPs with standard antibiotic therapy enhanced antibacterial efficacy and significantly reduced inflammation and apoptosis markers, indicating a synergistic effect.

### 4.1. Efficacy of EuChiNPs

The encapsulation of eugenol in ChiNPs provided several advantages, including improved stability, bioavailability, and controlled release. DLS and TEM analysis confirmed the nanoparticles’ uniform size distribution and stability, which are essential factors for targeted drug delivery. ATR‐FTIR spectra further validated the successful loading of eugenol into ChiNPs.

Our results align with previous studies demonstrating the therapeutic potential of eugenol. For instance, Elbestawy et al. reported that eugenol exhibits antibacterial and anti‐inflammatory effects against *H. pylori* infections although their study did not utilize nanoparticle delivery systems. By incorporating eugenol into ChiNPs, we observed enhanced therapeutic efficacy, particularly when combined with antibiotics [15, 21].

### 4.2. Reduction in Inflammatory Markers

In *H. pylori* infection, inflammatory cytokines such as IL‐1β, IL‐8, and TNF‐α play critical roles in gastric mucosal damage. Our study demonstrated that the combination of EuChiNPs and standard antibiotics significantly reduced the expression of these markers compared with antibiotics alone. These findings suggest that EuChiNPs can modulate inflammatory pathways, thereby reducing the severity of infection‐induced gastric inflammation. The reduction in inflammatory markers aligns with studies highlighting eugenol’s anti‐inflammatory properties. For example, Fayed et al. showed that the coadministration of chitosan‐based nanoparticles and antibiotics decreased inflammation in *H. pylori*‐infected models. Similarly, Fan et al. reported elevated levels of IL‐1β and TNF‐α in *H. pylori*‐infected patients, further emphasizing the significance of targeting these markers in therapeutic strategies [21–24]. Magdalena U. et al. indicated that eugenol exerts its anti‐inflammatory properties by inhibiting Raf/MEK/ERK1/2/p47‐phosphorylation pathway in neutrophils and suppressing pro‐inflammatory mediators such as IL‐1β, IL‐8, and TNF‐α. Its anti‐inflammatory activity is primarily attributed to neutrophil and macrophage chemotaxis inhibition. Additionally, eugenol can reduce inflammation by inhibiting the production of inflammatory neurotransmitters such as prostaglandins and leukotrienes [24].

### 4.3. Apoptosis Modulation and Gastric Mucosa Protection

Apoptosis is a hallmark of *H. pylori*‐induced gastric damage. Western blot analysis in our study revealed a significant reduction in caspase‐3 and caspase‐7 levels in the EuChiNPs + antibiotics group, suggesting reduced apoptotic activity. Histological analyses corroborated these findings, showing improved mucosal integrity and reduced bacterial density in the EuChiNPs + antibiotics group compared with other treatment groups. These results are consistent with prior research. Sarkar et al. demonstrated that eugenol induces apoptosis in cancer cells by downregulating proapoptotic proteins [12], while ChiNPs have been shown to enhance mucosal protection. Our findings extend this knowledge by highlighting the dual role of EuChiNPs in bacterial eradication and mucosal preservation. Studies have shown that EuChiNPs induce apoptosis in C6 glimoma cells in animal models [19]. Additionally, a separate study investigating the mechanism of action of eugenol has indicated that this compound works through various pathways, including upregulation of tumor suppressor genes, apoptotic inducer proteins, and downregulation of apoptotic proteins, oncogenes, transcription factors, and cell survival proteins [19, 20, 25].

### 4.4. Implications for Reducing Antibiotic Resistance

The rise of antibiotic resistance in *H. pylori* treatment has necessitated the exploration of alternative and complementary therapies. Our study highlights the potential of EuChiNPs as an adjunct to standard antibiotics, allowing for reduced antibiotic dosages without compromising therapeutic efficacy. This approach could mitigate the risk of developing resistance while maintaining high eradication rates.

Recent studies, such as those by Henriques et al., have emphasized the role of chitosan‐based formulations in improving the retention and efficacy of antibiotics against resistant *H. pylori* strains. Cheng‐Jung Yao et al. developed chitosan–loaded amoxicillin nanoparticles and successfully potentiated the treatment of *H. pylori* in a mouse model. Chitosan has a mucoadhesive property that enables it to penetrate the layers of the gastric epithelium’s mucus, gradually releasing amoxicillin and increasing the drug’s effect. Our findings add to this body of evidence, suggesting that combining EuChiNPs and antibiotics can be a viable strategy for overcoming resistance challenges [26–28].

Previous studies have reported the antibacterial and anti‐inflammatory properties of eugenol, as well as the therapeutic potential of chitosan and chitosan‐based nanoparticles against various bacterial infections [29–31]. However, most of these investigations have been limited to in vitro models or have evaluated either free eugenol or chitosan formulations without comprehensive in vivo validation against *H. pylori*. In contrast, the present study provides a direct in vivo comparison by demonstrating that EuChiNPs significantly reduce bacterial colonization, attenuate inflammatory responses, and modulate apoptotic markers in a rat model of *H. pylori* infection. Moreover, the observed synergistic effect when EuChiNPs were combined with standard antibiotics further distinguishes our findings from previous reports and highlights the added therapeutic value of nanoencapsulation for enhancing the efficacy of eugenol.

### 4.5. Limitations and Future Directions

Despite the promising results of this study regarding the efficacy of EuChiNPs in the treatment of *H. pylori* infection, several limitations must be considered when interpreting the findings. First, the study was conducted on a single strain of *H. pylori* using an animal model (rats). However, there are substantial structural and functional differences between rodents and humans in terms of physiology, immune responses, gastrointestinal architecture, and gut microbiota, which may influence drug bioavailability, local effects, immune modulation, and apoptotic pathways—potentially leading to different therapeutic outcomes. For example, differences in gastric pH, digestive enzyme composition, mucosal structure, and microbial diversity can affect the absorption and action of the nanoparticle formulation. Moreover, molecular pathways involved in inflammation and apoptosis may function differently across species, thus requiring caution when extrapolating these results to human populations. Additionally, the duration of the study was relatively short, and long‐term effects such as recurrence of infection, alterations in gut microbiota composition, and the development of drug resistance were not assessed. Although the findings showed reductions in inflammatory and apoptotic markers, more detailed molecular studies—including gene expression and signaling pathway analysis—are needed to fully elucidate the therapeutic mechanisms of EuChiNPs. Therefore, future research should include studies on various *H. pylori* strains, extended observation periods, and ultimately, well‐designed human clinical trials to evaluate the efficacy, safety, and bioavailability of this formulation. Furthermore, investigating the long‐term effects of EuChiNPs on the human microbiota and gastric mucosal immunity will be crucial for comprehensively assessing the safety and therapeutic value of this novel treatment strategy. The release profile, swelling index, drug kinetic models, cumulative drug release, and thermal stability assessments were not performed in this study and will be addressed in future research.

## 5. Conclusion

In summary, our study demonstrates that EuChiNPs exert potent therapeutic effects against *H. pylori* infection in a rat model. EuChiNPs significantly reduced bacterial colonization and protected gastric mucosa, while simultaneously downregulating key inflammatory mediators (IL‐1β, IL‐8, and TNF‐α) and apoptotic markers (caspase‐3 and caspase‐7). Notably, when combined with standard antibiotics, EuChiNPs produced a synergistic effect, leading to superior bacterial eradication, marked mucosal healing, and enhanced suppression of inflammation and apoptosis compared with antibiotics alone. These findings not only validate EuChiNPs as a promising adjunctive therapeutic strategy but also highlight their potential to lower antibiotic dosage, mitigate resistance, and improve treatment outcomes in *H. pylori*‐associated diseases. Future clinical studies are warranted to translate these preclinical findings into effective therapies for human patients.

## Disclosure

All authors provided feedback on previous versions of the manuscript and approved the final version.

## Conflicts of Interest

The authors declare no conflicts of interest.

## Author Contributions

All authors contributed to the study conception and design. Study design, material preparation, data collection, and analysis were carried out by B.M. The initial draft of the manuscript was written by M.J.T. and M.O., and experiments were conducted by B.M., M.O., A.T., F.N., A.A., and E.Z.

## Funding

This study was funded by the Fasa University of Medical Science, code 400227.

## Data Availability

All data will be available on reasonable request from the corresponding author.
